# Associations Between Nutritional Deficits and Physical Performance in Community-Dwelling Older Adults

**DOI:** 10.3389/fnut.2021.771470

**Published:** 2021-11-11

**Authors:** Wan-Hsuan Lu, Kelly Virecoulon Giudici, Yves Rolland, Sophie Guyonnet, Jean-François Mangin, Bruno Vellas, Philipe de Souto Barreto

**Affiliations:** ^1^Gerontopole of Toulouse, Institute of Ageing, Toulouse University Hospital (CHU Toulouse), Toulouse, France; ^2^Maintain Aging Research Team, CERPOP, INSERM, Université Paul Sabatier, Toulouse, France; ^3^CATI Multicenter Neuroimaging Platform, Neurospin, CEA, Gif-sur-Yvette, France; ^4^Université Paris-Saclay, CEA, CNRS, Neurospin, Baobab, Gif-sur-Yvette, France

**Keywords:** vitamin D, homocysteine, omega-3 fatty acids, physical performance, inflammation

## Abstract

**Background:** Whether multiple nutritional deficiencies have a synergic effect on mobility loss remains unknown. This study aims to evaluate associations between multi-nutritional deficits and physical performance evolution among community-dwelling older adults.

**Methods:** We included 386 participants from the Multidomain Alzheimer Preventive Trial (MAPT) (75.6 ± 4.5 years) not receiving omega-3 polyunsaturated fatty acid (PUFA) supplementation and who had available data on nutritional deficits. Baseline nutritional deficits were defined as plasma 25 hydroxyvitamin D <20 ng/ml, plasma homocysteine >14 μmol/L, or erythrocyte omega-3 PUFA index ≤ 4.87% (lower quartile). The Short Physical Performance Battery (SPPB), gait speed, and chair rise time were used to assess physical performance at baseline and after 6, 12, 24, 36, 48, and 60 months. We explored if nutrition-physical performance associations varied according to the presence of low-grade inflammation (LGI) and brain imaging indicators.

**Results:** Within-group comparisons showed that physical function (decreased SPPB and gait speed, increased chair rise time) worsened over time, particularly in participants with ≥2 nutritional deficits; however, no between-group differences were observed when individuals without deficit and those with either 1 or ≥2 deficits were compared. Our exploratory analysis on nutritional deficit-LGI interactions showed that, among people with ≥2 deficits, chair rise time was increased over time in participants with LGI (adjusted mean difference: 3.47; 95% CI: 1.03, 5.91; *p* = 0.017), compared with individuals with no LGI.

**Conclusions:** Accumulated deficits on vitamin D, homocysteine, and omega-3 PUFA were not associated with physical performance evolution in older adults, but they determined declined chair rise performance in subjects with low-grade inflammation.

**Clinical Trial Registration:** [https://clinicaltrials.gov/ct2/show/NCT00672685], identifier [NCT00672685].

## Introduction

Decline in physical performance, as measured by lower extremity function, often marks the early stage of disability in older age (1, 2). It is crucial to identify modifiable factors, such as nutritional risk factors, and their underlying biological mechanisms leading to impaired mobility in older individuals. Indeed, several blood-based nutritional markers, such as homocysteine, vitamin D, and omega-3 polyunsaturated fatty acids (PUFAs), have gradually become the focus of research and clinical interventions (3, 4). Hyperhomocysteinemia (HHcy) has been associated with faster physical impairment, such as in walking test and chair rise test, in several longitudinal studies (5–7). Vitamin D deficiency has been cross-sectionally associated with poor physical performance (8–10); however, similar associations were not discovered in longitudinal studies (11, 12). Although the literature on omega-3 PUFAs is mixed, some studies have found increased omega-3 PUFAs was associated with low risk of mobility disability (13), poor Short Physical Performance Battery (SPPB) score, and slower gait speed over time (14).

Considering that the accumulation of deficits can be related with the ability of an individual to respond to stressors (15), it is possible that combined deficiencies in homocysteine, vitamin D, and omega-3 PUFAs would work synergistically to determine physical performance over time. This concept had been supported by the findings of a previous study, which have indicated that an increasing number of nutritional deficits were associated with faster cognitive decline (16). In another study, the nutritional index, which was constructed with 41 nutrition-related parameters from anthropometric, plasma, and nutrient intake measurements, had shown a stronger prediction of frailty and mortality risk compared with single nutritional parameters separately (15). Furthermore, there is a lack of studies investigating underlying mechanisms behind multi-nutritional deficits and physical impairment. Indeed, several physiological deteriorations that drive age-related mobility loss (17), including changes in the central nervous system (CNS) and chronic inflammation, have shown intimate associations with these nutritional markers (18, 19). For instance, HHcy can promote inflammation (20). Higher circulating levels of omega-3 were associated with larger hippocampal volume (21); on the other hand, smaller brain volumes were observed in people with low vitamin D status (22). Therefore, exploring the interactions between nutritional deficits and physiological alterations (i.e., chronic inflammation and CNS changes) might allow us to understand better their shared biological pathways leading to mobility decline.

The main objective of this study was to investigate the associations between multi-nutritional deficits (i.e., vitamin D deficiency, HHcy, and low omega-3 PUFA index) and physical performance in community-dwelling older adults over 5 years. In addition, we explored if the nutrition-physical performance associations varied according to the presence of low-grade inflammation (LGI) and brain imaging indicators.

## Methods

### Design and Ethical Statement

This observational study used data from Multidomain Alzheimer Preventive Trial (MAPT), whose details have been described in previous publications (23, 24). Briefly, MAPT was a multicenter, 3-year randomized controlled trial that aimed to evaluate the protective effect of omega-3 PUFA supplementation and multidomain lifestyle interventions (exercise advice, cognitive training, and nutritional counseling), combined or alone, on cognitive decline in community-dwelling older adults (23). No significant effect of the interventions on cognitive function (24) or muscle strength (25) was found over 3 years. In this secondary analysis, 5-year follow-up data (3-year intervention plus an additional 2-year observation period after the end of interventions) were retrieved. The MAPT study was registered at ClinicalTrials.gov (no: NCT00672685), was approved by the French Ethical Committee located in Toulouse (CPP SOOM II), and was authorized by the French Health Authority. All the participants had signed informed consent.

### Study Population

The MAPT study enrolled 1680 dementia-free adults aged ≥70 years, recruited from 13 memory centers in France and Monaco between 2008 to 2011, presenting at least one of the following conditions: spontaneous memory complaint, limitations in one instrumental Activities of daily living (such as disability in using telephone and transportation.), or slow gait speed (≤0.8 m/s). In this study, 840 subjects who received the intervention of omega-3 supplementation and 454 subjects without nutritional markers measurement at baseline were not included; we finally considered data from 386 subjects into this study.

### Measures

#### Definition of Nutritional Deficits

Three nutritional markers were used to define nutritional deficits: plasma 25-hydroxyvitamin D [25(OH)D], plasma homocysteine, and erythrocyte membrane omega-3 PUFA concentration. Details of nutritional marker assessment are described in Supplementary Materials. Nutritional deficits were determined at baseline according to the clinical cutoffs below: (1) vitamin D deficiency, if 25(OH)D <20 ng/ml (26); (2) HHcy, if homocysteine >14 μmol/L (27); (3) low omega-3 PUFA index, defined as omega-3 index (28) [% docosahexaenoic acid (DHA) + % eicosapentaenoic acid (EPA)] below the lower quartile of study population (≤4.87%) (16). The participants were then classified into three groups based on the counting of nutritional deficits: no deficit, 1 deficit, and ≥2 deficits.

#### Physical Performance

Three outcomes of physical performance were evaluated in this study: 4-m usual pace gait speed (in m/s), 5-repetition maximal chair rise time (in s), and Short Physical Performance Battery (SPPB) (29) score. The SPPB consisted of a walk test, a chair rise test, and a standing balance test with three challenging positions; each component was scored ranging from 0 (inability to complete the test) to 4 (best performance). The overall SPPB score was calculated by summing the three component results (ranging from 0 to 12, higher score indicates better performance) (29). All the measurements were assessed at baseline, and after 6, 12, 24, 36, 48, and 60 months of follow-up.

#### Low-Grade Inflammation (LGI)

In this study, 293 of the 386 participants had C-reactive protein (CRP) measured at baseline, 6- and 12-month visits, using immunoturbidity according to standard protocols. LGI (dichotomous variable) was defined as having at least two consecutively high CRP values (3–10 mg/L) between baseline and the 12-month visit, according to previous studies (30–32). Participants we could not categorize as LGI or non-LGI (e.g., people with CRP value >10 mg/L in the intermediate 6-month measurement) were excluded. Finally, we included 267 participants in the exploratory analysis.

#### Magnetic Resonance Imaging (MRI) Variables

Several MRI variables that had been reported to be associated with impaired mobility (18, 33, 34) were retrieved: total gray matter volume (cm^3^), hippocampal volume (mm^3^) and white matter hyperintensity (WMH) volume (cm^3^). Total intracranial volume (TICV) was also collected for model adjustment. The acquisition protocol for brain MRI has been detailed elsewhere (23) and in Supplementary Materials.

#### Confounders

Several confounding variables were selected: age, sex, MAPT intervention groups (i.e., multidomain intervention alone or placebo), level of education (ordinal), and body mass index (BMI; kg/m^2^). We also controlled the baseline physical activity status using a dichotomous variable (active or inactive) based on low physical activity component in the Fried's frailty criteria (35). In the analysis for MRI variables, adjusted models included the confounders mentioned above as well as TICV.

#### Statistical Analysis

Baseline characteristic comparisons across the nutritional deficit groups were performed by Chi-square test for categorical variables and ANOVA for continuous variables. Linear mixed-effect regressions, including a random effect at participant level and a random slope on time, were conducted to evaluate the cross-sectional and longitudinal associations between nutritional deficits and physical performance outcomes.

A series of exploratory analyses were conducted to explore the roles of LGI (among 267 subjects with available CRP data) and imaging markers (among 164 subjects with MRI measures) in the association between nutritional deficits and physical performance. We first performed logistic regressions to examine the association between nutritional deficits and LGI. Then, an interaction term by LGI and nutritional deficits was introduced into the same mixed-effect models for main analysis; only assessments of the outcomes performed at 12 months and after were considered for this analysis. For imaging markers, cross-sectional associations with nutritional deficits were tested by linear mixed-effect regressions (with random intercept for the center effect). Longitudinal analysis considering the interaction effect (MRI variable × nutritional deficits) on physical performance was examined by linear mixed-effect regressions (three-level nested model, with the participants nested into the center); for participants who received MRI scans at 6-month visit and 12-month visit, measurements of physical performances before MRI scans were excluded from the analysis. Statistical significance was defined as *p* < 0.05; the *p*-values of between-group mean differences are presented after false discovery rate correction (36). All the statistical analyses were performed with Statistical Analysis Software (SAS) version 9.4 (Cary, NC, USA).

## Results

Among the overall 386 participants, 21.8% (*n* = 84) had no nutritional deficit at baseline, 39.6% (*n* = 153) presented 1 deficit, and 38.6% (*n* = 149) presented ≥2 deficits. Participants with more nutritional deficits tended to be older and male, and to present with higher BMI (Table 1). At baseline, compared with those without any deficit, having ≥2 deficits was associated with longer chair rise time, i.e., poor chair rise performance (Table 2). No cross-sectional associations were found with SPPB score or gait speed.

**Table 1:** Baseline characteristics of the study population^1^.

	**Total population (*N* = 386)**	**Number of nutritional deficits^2^**			
		**No deficit** **(*N* = 84)**	**1 deficit** **(*N* = 153)**	**≥2 deficits** **(*N* = 149)**	***p*-value^3^**
Age (years)	75.6 (4.5)	74.8 (4.1)^a^	75.0 (4.3)^b^	76.7 (4.8)^a,b^	0.001
Sex (female)	263 (68.1%)	66 (78.6%)	111 (72.6%)	86 (57.7%)	0.002
**MAPT groups**					
Multidomain intervention	191 (49.5%)	40 (47.6%)	77 (50.3%)	74 (49.7%)	0.922
Placebo	195 (50.5%)	44 (52.4%)	76 (49.7%)	75 (50.3%)	
**Education (*****n*** **=** **376)**					
No diploma or primary school certificate	80 (21.3%)	13 (15.9%)	30 (19.9%)	37 (25.9%)	0.407
Secondary education	122 (32.4%)	28 (34.1%)	47 (31.1%)	47 (32.9%)	
High school diploma	58 (15.4%)	12 (14.6%)	29 (19.2%)	17 (11.9%)	
University level	116 (30.9%)	29 (35.4%)	45 (29.8%)	42 (29.3%)	
Body mass index (kg/m^2^) (*n* = 385)	26.1 (3.9)	25.1 (4.0)^a^	25.9 (3.9)	26.8 (3.8)^a^	0.003
CDR status: 0.5 (mild cognitive impairment)	155 (40.2%)	27 (32.1%)	64 (41.8%)	64 (43.0%)	0.234
Physical status: inactive (*n* = 384)	53 (13.8%)	5 (6.0%)	22 (14.5%)	26 (17.5%)	0.051
**Nutritional risk factors**					
Vitamin D (ng/ml)	23.2 (12.5)	30.8 (12.2)^a,b^	25.2 (12.7)^a,c^	16.9 (8.8)^b,c^	<0.001
Deficiency (<20 ng/ml)	163 (42.2%)	0 (0%)	57 (37.3%)	106 (71.1%)	<0.001
Homocysteine (μmol/L)	15.64 (5.27)	11.32 (1.41)^a,b^	15.31 (4.88)^a,c^	18.41 (5.32)^b,c^	<0.001
Hyperhomocysteinemia (>14 μmol/L)	220 (57.0%)	0 (0%)	85 (55.6%)	135 (90.6%)	<0.001
Omega-3 index (%)	5.86 (1.44)	6.61 (1.13)^a^	6.21 (1.25)^b^	5.08 (1.41)^a,b^	<0.001
≤ 4.87 (lower quartile)	98 (25.4%)	0 (0%)	11 (7.2%)	87 (58.4%)	<0.001
**Physical performance**					
SPPB score, 0–12 (*n* = 378)	10.7 (1.7)	10.9 (1.5)	10.7 (1.7)	10.5 (1.8)	0.156
Gait speed (m/s) (*n* = 383)	1.08 (0.25)	1.10 (0.25)	1.08 (0.25)	1.06 (0.25)	0.590
Chair rise time (s) (*n* = 367)	11.6 (3.9)	10.4 (2.4)^a^	11.6 (3.9)	12.3 (4.5)^a^	0.004

1 *Values presented in number (%) for categorical variables or mean (standard deviation) for continuous variables; CDR, clinical dementia rating scale; MAPT, Multidomain Alzheimer Preventive Trial; SPPB, Short Physical Performance Battery*.

2 *Cutoff of nutritional deficits: Vitamin D <20 ng/ml, homocysteine>14 μmol/L, omega-3 index ≤ lower quartile (≤4.87%)*.

3 *P-value based on ANOVA or Chi-square test across groups*;

a,b,c *same letters indicate significant differences between groups (p <0.05)*.

**Table 2:** Linear mixed-effect regressions examining cross-sectional associations between nutritional deficits^a^ and physical performance at baseline.

	**Unadjusted model**			**Adjusted model^b^**		
	**β**	**95% CI**	***p*-value**	**β**	**95% CI**	***p*-value**
**Outcome: SPPB score (0–12)**						
Nutritional deficits						
No deficit	Ref.	–	–	Ref.	–	–
1 deficit	−0.20	−0.60, 0.19	0.318	−0.16	−0.53, 0.21	0.391
≥2x deficits	−0.59	−0.99, −0.20	0.004	−0.33	−0.71, 0.05	0.089
**Outcome: gait speed (m/s)**						
Nutritional deficits						
No deficit	Ref.	–	–	Ref.	–	–
1 deficit	−0.01	−0.07, 0.05	0.703	−0.01	−0.06, 0.05	0.906
≥2 deficits	−0.06	−0.12, −0.01	0.041	−0.02	−0.08, 0.04	0.525
**Outcome: chair rise time (s)**						
Nutritional deficits						
No deficit	Ref.	–	–	Ref.	–	–
1 deficit	0.70	−0.22, 1.61	0.137	0.60	−0.28, 1.49	0.182
≥2 deficits	1.58	0.66, 2.50	0.001	0.99	0.07, 1.90	0.036

*CI, confidence interval; Ref, reference group; SPPB, Short Physical Performance Battery*.

a *Cutoff of nutritional deficits: vitamin D <20 ng/ml, homocysteine > 14 μmol/L, omega-3 index ≤ lower quartile (4.87%)*.

b *Adjustments for age, sex, Multidomain Alzheimer Preventive Trial (MAPT) groups, education, body mass index, and physical activity status*.

After 5 years of follow-up, decreased SPPB score and gait speed, and increased chair rise time were observed among the participants with ≥ 2 nutritional deficits (Table 3). *P*-values for linear trend for within-group change in physical performance outcomes were all significant (*p* for trend <0.001). However, no significant between-group differences were discovered for the changes in SPPB, gait speed, and chair rise time when individuals without deficit and those with either 1 or ≥2 deficits were compared (Table 3).

**Table 3:** Linear mixed-effect regressions examining variation in physical performance over 5 years according to nutritional deficits^a^.

	**Within-group 5-year change from baseline β (95% CI); *p*-value**	***P* for trend**	**Between-group difference**	
			**Unadjusted model β (95% CI); *p*-value^c^**	**Adjusted model^b^ β (95% CI); *p*-value^c^**
**Outcome: SPPB score (0–12)**				
Nutritional deficits		<0.001		
No deficit,	−0.43 (−0.86, 0.01); 0.057		Ref.	Ref.
1 deficit,	−0.47 (−0.84, −0.11); 0.011		−0.05 (−0.62, 0.52); 0.871	−0.03 (−0.59, 0.53); 0.921
≥2 deficits)	−0.80 (−1.17, −0.42); <0.001		−0.37 (−0.95, 0.20); 0.411	−0.23 (−0.80, 0.33); 0.843
**Outcome: gait speed (m/s)**				
Nutritional deficits		<0.001		
No deficit,	−0.09 (−0.16, −0.02); 0.010		Ref.	Ref.
1 deficit,	−0.05 (−0.11, 0.01); 0.052		0.03 (−0.05, 0.12); 0.879	0.03 (−0.06, 0.11); 0.813
≥2 deficits)	−0.08 (−0.14, −0.03); 0.005		0.01 (−0.08, 0.09); 0.879	−0.01 (−0.10, 0.08); 0.813
**Outcome: chair rise time (s)**				
Nutritional deficits		<0.001		
No deficit,	0.77 (−0.08, 1.62); 0.075		Ref.	Ref.
1 deficit,	0.46 (−0.24, 1.17); 0.198		−0.31 (−1.41, 0.80); 0.585	−0.18 (−1.26, 0.90); 0.739
≥2 deficits)	1.13 (0.39, 1.86); 0.003		0.36 (−0.77, 1.48); 0.585	0.40 (−0.71, 1.50); 0.739

*CI, confidence interval; Ref, reference group; SPPB, Short Physical Performance Battery*.

a *Cutoff of nutritional deficits: vitamin D <20 ng/ml, homocysteine >14 μmol/L, omega-3 index ≤ lower quartile (≤4.87%)*.

b *Adjustments for age, sex, Multidomain Alzheimer Preventive Trial (MAPT) groups, education, body mass index, physical activity status, and time interactions*.

c *P-value adjusted for multiple comparisons using the Benjamini-Hochberg procedure*.

In the logistic regression for LGI and nutritional deficits, people with ≥2 deficits had higher likelihood of having LGI (adjusted OR = 2.53; 95% CI: 1.01 to 6.33; *p* = 0.006; Supplementary Table 1), compared with those without deficits. Significant interaction effects by LGI and nutritional deficits on chair rise time were observed in the linear mixed-effect regression. Among people with ≥2 deficits, the adjusted mean difference in chair rise time over 5 years between those with and without LGI (reference group) was 3.47 s (95% CI: 1.03, 5.91; *p* = 0.017), indicating that LGI reinforced the impact of ≥2 deficits on worsening chair rise performance (Supplementary Table 2). On the other hand, no association between imaging markers and nutritional deficits was found (Supplementary Table 3). There was no evidence of any significant interaction between each imaging marker and nutritional deficits on physical performances in the linear mixed-effect models (Supplementary Table 4).

## Discussion

To our knowledge, this is the first study to investigate the associations between accumulated nutritional deficits and physical performance in community-dwelling older adults. We discovered that presenting two or more nutritional deficits (i.e., vitamin D deficiency, HHcy, and low omega-3 PUFA index) was cross-sectionally associated with poor chair rise performance at baseline. We did not observe associations of combined nutritional deficits with mobility decline over 5 years; however, our exploratory analysis found that the association of nutritional deficits with chair rise performance could vary according to LGI status, with a more pronounced increase in chair rise time (worse performance; 0.69 s more per year) among older adults with ≥2 deficits and LGI compared with their non-LGI counterparts.

The relationship between the nutritional markers investigated in our study and physical performance has mixed findings in the literature (8–13). In this study, although the between-group differences did not reach significance, within-group changes for all the three physical performance outcomes showed higher overtime declines as the number of deficits increased (*p* for trend <0.001). Noteworthy, after the 5-year follow-up, more than half of the participants with ≥2 nutritional deficits became octogenarians whose mobility tends to decline faster than in younger people (17). On the other hand, our exploratory analysis found that LGI, an important mechanism implicated in both aging (37) and mobility disability (17, 38), contributed to this accelerated decline of physical performance in older individuals with combined nutritional deficits. This finding suggests that both the presence of nutritional deficits and chronic inflammation contribute to physical impairment. Indeed, omega-3 PUFAs and HHcy had been proposed to affect mobility outcomes through inflammatory pathway. Omega-3 PUFAs can suppress chronic inflammation, further inhibiting muscle catabolism (39); HHcy can lead to inflammation by causing reactive oxygen species accumulation and pro-inflammatory cytokine secretion (4, 20). Although vitamin D is well-known for its metabolic roles in muscle synthesis and bone formation (3), the recent evidence had suggested it has immunomodulatory effects by regulating both innate and adaptive immunity (40). On the other hand, it is plausible that LGI status is independent of the presence of nutritional deficits, but that their joint effect enhances the detrimental impact on physical function. For example, accelerated muscle catabolism caused by inflammation (41), combined with muscle weakness caused by vitamin D deficiency (10), can lead to faster decline in overall muscle function and physical performance.

Our cross-sectional and exploratory analyses only observed significant associations between nutritional deficits and chair rise performance, suggesting nutritional deficits would affect physical performance through a muscle quality-related mechanism rather than changes in the central nervous system. This is also supported by our findings related to MRI indicators, which showed no significant interaction between brain volumes and nutritional deficits on changes in physical performance over time. Compared with gait speed, a functional vital sign (42) relying on complex movement controls with executive function involved (43), chair rise test is a more specific measure of muscle function (44), strongly determined by muscle mass and power in older adults (45, 46). Another possible explanation for the limited findings on change in SPPB is that only a few participants of this study had mobility limitation at baseline, with 6% having SPPB ≤ 7 (1) and about 20% having SPPB ≤ 9 (47). Although it is possible that people who started having mobility limitations would decline faster in mobility (48), the associations of nutritional deficits with mobility limitation in mobility-impaired individuals require further investigations.

A number of strengths should be mentioned in our study. We evaluated multiple nutritional deficits, assessed by three blood-based biomarkers, and several measures of physical performance in older adults over 5 years. In addition, we explored the potential role of inflammatory and neuroimaging markers in nutrition-physical performance associations. However, some limitations are worth mentioning. First, this is an observational study with data retrieved from a randomized controlled trial. Even though MAPT multidomain intervention did not affect physical performance (25), our results need to be interpreted cautiously, since the exercise advice and nutritional counseling part of the multidomain intervention could have modified the nutritional markers overtime. In order to minimize this bias, MAPT group allocation was added as a confounder in the models. Residual confounding may not be excluded, since some other potential confounders, such as nutritional supplementation (except for omega-3 PUFAs), inadequate dietary protein intake, and smoking and drinking habits (12, 49), were not available. Finally, the MAPT study enrolled a sample of community-dwelling older adults at risk of cognitive decline, which might affect the generalizability of our findings to other populations.

To conclude, this study did not observe prospective associations between combined nutritional deficits (vitamin D deficiency, HHcy, and low omega-3 index) and overtime mobility decline in community-dwelling older adults. However, different trajectories of chair rise performance were observed among people with two or more deficits, once the presence of chronic, low-grade inflammation was considered. Future studies that will investigate nutritional deficits and physical impairment focused on older adults with different conditions characterized by LGI, including subjects with mobility limitation, could shed light on this topic.

## Data Availability Statement

Data described in the article, code book, and analytic code will be made available upon request pending application and approval. Requests to access these datasets should be directed to Wan-Hsuan Lu, wan-hsuan.lu1@univ-tlse3.fr.

## Ethics Statement

The studies involving human participants were reviewed and approved by French Ethical Committee located in Toulouse (CPP SOOM II). The patients/participants provided their written informed consent to participate in this study.

## MAPT/DSA Group

MAPT study group: principal investigator: Bruno Vellas (Toulouse); coordination: Sophie Guyonnet; project leader: Isabelle Carrié; CRA: Lauréane Brigitte; investigators: Catherine Faisant, Françoise Lala, Julien Delrieu, and Hélène Villars; psychologists: Emeline Combrouze, Carole Badufle, and Audrey Zueras; methodology, statistical analysis, and data management: Sandrine Andrieu, Christelle Cantet, and Christophe Morin; multidomain group: Gabor Abellan Van Kan, Charlotte Dupuy, and Yves Rolland (physical and nutritional components), Céline Caillaud, Pierre-Jean Ousset (cognitive component), and Françoise Lala (preventive consultation) (Toulouse). The cognitive component was designed in collaboration with Sherry Willis from the University of Seattle, and Sylvie Belleville, Brigitte Gilbert, and Francine Fontaine from the University of Montreal. Co-investigators in associated centers: Jean-François Dartigues, Isabelle Marcet, Fleur Delva, Alexandra Foubert, and Sandrine Cerda (Bordeaux); Marie-Noëlle-Cuffi, Corinne Costes (Castres); Olivier Rouaud, Patrick Manckoundia, Valérie Quipourt, Sophie Marilier, and Evelyne Franon (Dijon); Lawrence Bories, Marie-Laure Pader, Marie-France Basset, Bruno Lapoujade, Valérie Faure, Michael Li Yung Tong, Christine Malick-Loiseau, and Evelyne Cazaban-Campistron (Foix); Françoise Desclaux and Colette Blatge (Lavaur); Thierry Dantoine, Cécile Laubarie-Mouret, Isabelle Saulnier, Jean-Pierre Clément, Marie-Agnès Picat, Laurence Bernard-Bourzeix, Stéphanie Willebois, Iléana Désormais, and Noëlle Cardinaud (Limoges); Marc Bonnefoy, Pierre Livet, Pascale Rebaudet, Claire Gédéon, Catherine Burdet, and Flavien Terracol (Lyon), Alain Pesce, Stéphanie Roth, Sylvie Chaillou, and Sandrine Louchart (Monaco); Kristel Sudres, Nicolas Lebrun, and Nadège Barro-Belaygues (Montauban); Jacques Touchon, Karim Bennys, Audrey Gabelle, Aurélia Romano, Lynda Touati, Cécilia Marelli, and Cécile Pays (Montpellier); Philippe Robert, Franck Le Duff, Claire Gervais, and Sébastien Gonfrier (Nice); Yannick Gasnier, Serge Bordes, Danièle Begorre, Christian Carpuat, Khaled Khales, Jean-François Lefebvre, Samira Misbah El Idrissi, Pierre Skolil, and Jean-Pierre Salles (Tarbes). MRI group: Carole Dufouil (Bordeaux); Stéphane Lehéricy, Marie Chupin, Jean-François Mangin, and Ali Bouhayia (Paris); Michèle Allard (Bordeaux); Frédéric Ricolfi (Dijon); Dominique Dubois (Foix); Marie Paule Bonceour Martel (Limoges); François Cotton (Lyon); Alain Bonafé (Montpellier); Stéphane Chanalet (Nice); Françoise Hugon (Tarbes); Fabrice Bonneville, Christophe Cognard, and François Chollet (Toulouse). PET scans group: Pierre Payoux, Thierry Voisin, Julien Delrieu, Sophie Peiffer, and Anne Hitzel, (Toulouse); Michèle Allard (Bordeaux); Michel Zanca (Montpellier); Jacques Monteil (Limoges); Jacques Darcourt (Nice). Medico-economics group: Laurent Molinier, Hélène Derumeaux, and Nadège Costa (Toulouse). Biological sample collection: Bertrand Perret, Claire Vinel, and Sylvie Caspar-Bauguil (Toulouse). Safety management: Pascale Olivier-Abbal.

DSA group: Sandrine Andrieu, Christelle Cantet, and Nicola Coley.

## Author Contributions

BV conceived the MAPT study. W-HL and PSB designed current research. J-FM managed image data. W-HL performed statistical analysis, drafted the manuscript, and had a primary responsibility for the final content. All authors contributed to the article and approved the submitted version.

## Funding

This study was performed in the context of the Inspire Program, a research platform supported by grants from the Region Occitanie/Pyrénées-Méditerranée (Reference number: 1901175) and the European Regional Development Fund (ERDF) (Project number: MP0022856). The MAPT study was supported by grants from the Gérontopôle of Toulouse, the French Ministry of Health (PHRC 2008, 2009), Pierre Fabre Research Institute (manufacturer of the omega-3 supplement), ExonHit Therapeutics SA, and Avid Radiopharmaceuticals Inc. The promotion of this study was supported by the University Hospital Center of Toulouse. The data sharing activity was supported by the Association Monegasque pour la Recherche sur la maladied'Alzheimer's (AMPA) and the INSERM-University of Toulouse III UMR 1295 Unit.

## Conflict of Interest

The authors declare that the research was conducted in the absence of any commercial or financial relationships that could be construed as a potential conflict of interest.

## Publisher's Note

All claims expressed in this article are solely those of the authors and do not necessarily represent those of their affiliated organizations, or those of the publisher, the editors and the reviewers. Any product that may be evaluated in this article, or claim that may be made by its manufacturer, is not guaranteed or endorsed by the publisher.
