# Supplementary material for: Associations Between Nutritional Deficits and Physical Performance in Community-Dwelling Older Adults
Source: Front Nutr. 2021 Nov 11;8:771470. doi: 10.3389/fnut.2021.771470 (PMC8632557; doi:10.3389/fnut.2021.771470)
Supplement: Supplementary file 1 [file Data_Sheet_1.docx]

Supplementary Material

# Methods

## *Nutritional marker measurement*

Total plasma 25-hydroxyvitamin D ([25(OH)D]) (D3 and D2 forms(1)) was assessed using an electro-chemiluminescence binding assay (Cobas 8000; Roche). In brief, the sample was denatured to release bound 25(OH)D from vitamin D binding protein (VDBP). The sample was then incubated with recombinant ruthenium-labelled VDBP to enable complex formation. Biotinylated Vitamin D was subsequently added and the entire complex became bound to a solid phase by the interaction of biotin and streptavidin-coated microparticles. Unbound substances were removed and then applied a voltage to the electrode that induced a chemiluminescent and quantifiable emission. A concentration of 25(OH)D in ng/mL was derived against a standard curve.

Total plasma homocysteine was determined using an enzymatic cycling assay (Cobas, Roche)(2). Briefly, oxidized homocysteine was first reduced and then reacted with S-adenosylmethionine to form methionine and S-adenosylhomocysteine (SAH) in the presence of homocysteine S-methyltransferase. SAH was hydrolyzed into adenosine and homocysteine. The formed homocysteine was cycled back to the homocysteine conversion reaction, which served to amplify the detection signals. The formed adenosine was hydrolyzed into inosine and ammonia, which reacted with conversions of nicotinamide adenine dinucleotide (NADH) to NAD+, catalyzed by glutamate dehydrogenase. The homocysteine concentration in the sample was proportional to the amount of NADH converted to NAD+ (read at an absorbance of 340 nm) and measured in μmol/L against a standard curve.

Omega-3 polyunsaturated fatty acids (PUFA) assessment has been described previously(3,4). In short, lipids in erythrocyte membranes were extracted with a mixture of hexane and isopropanol after acidification. Margaric acid (Sigma) was added as an internal standard. Total lipid extracts were saponified and methylated. Fatty acid methyl esters (FAME) were extracted with pentane and analyzed by gas chromatography using an Agilent Technologies 6890N gas chromatograph with a split injector, a bonded silica capillary column and a flame ionization detector. Helium was used as a carrier gas. Identification of FAME was based on retention times obtained for FAME prepared from fatty acid standards. The area under the curve was determined using ChemStation software (Agilent) and results were expressed as a percent (%) of total fatty acids.

## *Magnetic resonance imaging (MRI) acquisition and analyses*

The MRI scan was performed among the subgroup of the MAPT population within the first 12 months of study enrollment, using a standardized protocol in each of the centers. In the present study, 164 participants underwent MRI scans (101 subjects were assessed between baseline and 6-month visit, and 63 subjects were evaluated between the 6- and the 12-month visit). Several MRI variables were retrieved: (1) total gray matter volume (cm^3^), using the 3D T1- weighted sequence derived by the SPM5 toolbox (fil.ion.ucl.ac.uk/spm); (2) hippocampal volume (mm^3^), measured by FreeSurfer software (version 5.3) (https://surfer. nmr.mgh.harvard.edu) and calculated as the mean of right and left hippocampal hemispheres; (3) volume of white matter hyperintensities (WMH) lesions (cm^3^), estimated by the automated White matter Hyperintensities Automated Segmentation Algorithm (WHASA)(5). Total intracranial volume (TICV, cm^3^) was retrieved for model adjustment using SPM5. The imaging quality of TICV, gray matter and WMH volume were first assessed, and data with poor or unreliable quality were excluded from the analysis.

## *References*

1. Holick MF. Vitamin D Status: Measurement, Interpretation, and Clinical Application [Internet]. Vol. 19, Annals of Epidemiology. Ann Epidemiol; 2009 [cited 2020 Aug 3]. p. 73–8. Available from: https://pubmed.ncbi.nlm.nih.gov/18329892/

2. Dou C, Xia D, Zhang L, Chen X, Flores P, Datta A, et al. Development of a novel enzymatic cycling assay for total homocysteine. In: Clinical Chemistry [Internet]. Oxford Academic; 2005 [cited 2020 Aug 3]. p. 1987–9. Available from: https://academic.oup.com/clinchem/article/51/10/1987/5629495

3. Legrand P, Schmitt B, Mourot J, Catheline D, Chesneau G, Mireaux M, et al. The consumption of food products from linseed-fed animals maintains erythrocyte omega-3 fatty acids in obese humans. Lipids [Internet]. 2010 Jan 11 [cited 2020 Aug 3];45(1):11–9. Available from: https://link.springer.com/article/10.1007/s11745-009-3376-5

4. Vellas B, Carrie I, Gillette-Guyonnet S, Touchon J, Dantoine T, Dartigues JF, et al. Mapt study: a multidomain approach for preventing Alzheimer’s disease: design and baseline data. J Prev Alzheimer’s Dis [Internet]. 2014 Jun;1(1):13–22. Available from: http://www.ncbi.nlm.nih.gov/pubmed/26594639

5. Samaille T, Fillon L, Cuingnet R, Jouvent E, Chabriat H, Dormont D, et al. Contrast-Based Fully Automatic Segmentation of White Matter Hyperintensities: Method and Validation. PLoS One [Internet]. 2012 Nov 12 [cited 2020 Oct 16];7(11). Available from: https://pubmed.ncbi.nlm.nih.gov/23152828/

# Supplementary Table 1. Logistic regression analysis evaluating the association between nutritional deficits^1^ and inflammation status.

|  | **Unadjusted model**  OR (95% CI); p-value | |  | **Adjusted model^2^**  OR (95% CI); p-value | |
| --- | --- | --- | --- | --- | --- |
|  | **1 deficit** | **≥2 deficits** |  | **1 deficit** | **≥2 deficits** |
| Lower-grade inflammation (LGI)^3^ | 0.82 (0.31, 2.20); 0.099 | 2.55 (1.06, 6.10); 0.003 |  | 0.81 (0.30, 2.23); 0.106 | 2.53 (1.01, 6.33); 0.006 |

OR, odds ratio of having lower-grade inflammation compared to the reference group (no nutritional deficit); CI, confidence interval.

^1^ Cutoff of nutritional deficits: 25-hydroxyvitamin D <20 ng/ml, homocysteine >14 μmol/L, omega-3 index ≤ lower quartile (≤4.87%).

^2^ Adjustment for age, sex, Multidomain Alzheimer Preventive Trial (MAPT) groups and body mass index.

^3^ LGI is defined as having at least two consecutively C-reactive protein values within 3 to 10 mg/L between baseline and the 12-month visit.

# Supplementary Table 2. Linear mixed-effect regressions examining variation in physical performance over five years according to nutritional deficits^1^ and inflammation status. Comparisons are performed between LGI and non-LGI individuals within each nutritional deficit group.

|  | **Within-group 5-year change from baseline**  β (95% CI); p-value | **Between-group difference** | |
| --- | --- | --- | --- |
|  |  | **Unadjusted model**  β (95% CI); p-value^3^ | **Adjusted model^2^**  β (95% CI); p-value^3^ |
| **Outcome: SPPB score (0-12)** |  |  |  |
| No LGI |  |  |  |
| No nutritional deficit | -0.67 (-1.28, -0.05); 0.033 | Ref. | Ref. |
| 1 deficit | -0.85 (-1.39, -0.31); 0.002 | Ref. | Ref. |
| ≥2 deficits | -0.49 (-1.18, 0.19); 0.156 | Ref. | Ref. |
| LGI^4^ |  |  |  |
| No nutritional deficit | -0.58 (-2.47, 1.31); 0.547 | 0.09 (-1.90, 2.08); 0.932 | 0.12 (-1.91, 2.14); 0.913 |
| 1 deficit | -1.05 (-2.75, 0.65); 0.225 | -0.20 (-1.98, 1.59); 0.932 | -0.10 (-1.91, 1.71); 0.913 |
| ≥2 deficits | -0.61 (-1.73, 0.50); 0.279 | -0.12 (-1.43, 1.19); 0.932 | -0.27 (-1.61, 1.06); 0.913 |
| **Outcome: Gait speed (m/s)** |  |  |  |
| No LGI |  |  |  |
| No nutritional deficit | -0.06 (-0.15, 0.02); 0.130 | Ref. | Ref. |
| 1 deficit | -0.08 (-0.15, 0.00); 0.043 | Ref. | Ref. |
| ≥2 deficits | -0.01 (-0.11, 0.08); 0.805 | Ref. | Ref. |
| LGI^4^ |  |  |  |
| No nutritional deficit | -0.26 (-0.52, 0.00); 0.050 | -0.20 (-0.47, 0.08); 0.240 | -0.20 (-0.47, 0.07); 0.230 |
| 1 deficit | -0.12 (-0.35, 0.11); 0.315 | -0.04 (-0.29, 0.20); 0.735 | -0.03 (-0.28, 0.21); 0.781 |
| ≥2 deficits | -0.19 (-0.34, -0.04); 0.015 | -0.18 (-0.36, 0.00); 0.150 | -0.17 (-0.35, 0.01); 0.181 |
| **Outcome: Chair rise time (s)** |  |  |  |
| No LGI |  |  |  |
| No nutritional deficit | 0.46 (-0.68, 1.60); 0.429 | Ref. | Ref. |
| 1 deficit | 1.35 (0.34, 2.37); 0.010 | Ref. | Ref. |
| ≥2 deficits | 0.63 (-0.67, 1.94); 0.339 | Ref. | Ref. |
| LGI^4^ |  |  |  |
| No nutritional deficit | 1.01 (-2.63, 4.64); 0.586 | 0.55 (-3.26, 4.36); 0.839 | 0.74 (-3.02, 4.50); 0.779 |
| 1 deficit | 1.01 (-2.13, 4.16); 0.526 | -0.34 (-3.65, 2.97); 0.839 | -0.46 (-3.72, 2.79); 0.779 |
| ≥2 deficits | 3.76 (1.65, 5.86); 0.001 | 3.12 (0.65, 5.60); 0.041 | 3.47 (1.03, 5.91); 0.017 |

CI, confidence interval; LGI, lower-grade inflammation; Ref.; reference group; SPPB, Short Physical Performance Battery.

^1^ Cutoff of nutritional deficits: 25-hydroxyvitamin D <20 ng/ml, homocysteine >14 μmol/L, omega-3 index ≤ lower quartile (≤4.87%).

^2^ Adjustments for age, sex, Multidomain Alzheimer Preventive Trial (MAPT) groups, education, body mass index, physical activity status, lower-grade inflammation (LGI) and interactions between LGI, nutritional deficits and time.

^3^ P-value adjusted for multiple comparisons using the Benjamini-Hochberg procedure.

^4^ LGI is defined as having at least two consecutively C-reactive protein values within 3 to 10 mg/L between baseline and the 12-month visit.

# Supplementary Table 3. Linear mixed-effect regressions evaluating the association between nutritional deficits1 and neuroimaging outcomes.

|  |  | **Unadjusted model^2^**  β (95% CI); p-value | | **Adjusted model^3^**  β (95% CI); p-value | |
| --- | --- | --- | --- | --- | --- |
|  | N | 1 deficit | ≥2 deficits | 1 deficit | ≥2 deficits |
| Gray matter volume (cm^3^) | 161 | 18.4 (-5.0, 41.8);  0.122 | 11.7 (-12.1, 35.4);  0.332 | -0.31 (-5.20, 4.58);  0.900 | 1.18 (-3.92, 6.27);  0.649 |
| Hippocampal volume (mm^3^) | 164 | -93.5 (-275.5, 88.6);  0.312 | -57.5 (-238.9, 123.9);  0.532 | -148.2 (-314.4, 18.0);  0.080 | -29.9 (-201.3, 141.5);  0.731 |
| WMH volume (cm^3^) | 151 | -0.11 (-3.52, 3.31);  0.951 | 0.58 (-2.90, 4.07);  0.741 | -0.44 (-3.57, 2.69);  0.781 | 0.16 (-3.14, 3.46);  0.925 |

CI, confidence interval; WMH, white matter hyperintensities.

^1^ Cutoff of nutritional deficits: 25-hydroxyvitamin D <20 ng/ml, homocysteine >14 μmol/L, omega-3 index ≤ lower quartile (≤4.87%).

^2^ Random slope on study center only.

^3^ Adjustments for age, sex, Multidomain Alzheimer Preventive Trial (MAPT) groups, education, body mass index and total intracranial volume.

# Supplementary Table 4. Linear mixed-effect regressions examining variation in physical performance over 5 years according to nutritional deficits^1^ and baseline brain volume.

|  | **Adjusted mean difference over 5 years per SD ﻿increment in brain volume^2^** | | | | | |
| --- | --- | --- | --- | --- | --- | --- |
|  | **No nutritional deficit** | | **1 deficit** | | **≥2 deficits** | |
|  | β (95% CI) | p for interaction^3^ | β (95% CI) | p for interaction^3^ | β (95% CI) | p for interaction^3^ |
| **Outcome: SPPB score (0-12)** |  |  |  |  |  |  |
| Gray matter volume | -0.09 (-0.76, 0.58) | 0.791 | 0.11 (-0.45, 0.68) | 0.698 | -0.25 (-1.02, 0.53) | 0.531 |
| Hippocampal volume | -0.21 (-0.95, 0.53) | 0.580 | 0.19 (-0.46, 0.85) | 0.562 | -0.03 (-0.61, 0.55) | 0.925 |
| WMH volume | -0.01 (-0.61, 0.61) | 0.999 | -0.33 (-0.96, 0.29) | 0.294 | -0.39 (-1.24, 0.46) | 0.366 |
| **Outcome: Gait speed (m/s)** |  |  |  |  |  |  |
| Gray matter volume | 0.10 (-0.01, 0.20) | 0.050 | 0.08 (-0.01, 0.16) | 0.063 | -0.03 (-0.14, 0.09) | 0.628 |
| Hippocampal volume | 0.01 (-0.11, 0.12) | 0.936 | 0.02 (-0.08, 0.12) | 0.709 | -0.02 (-0.11, 0.06) | 0.589 |
| WMH volume | -0.04 (-0.14, 0.06) | 0.456 | 0.01 (-0.09, 0.12) | 0.788 | 0.02 (-0.12, 0.16) | 0.770 |
| **Outcome: Chair rise time (s)** |  |  |  |  |  |  |
| Gray matter volume | 0.25 (-0.69, 1.19) | 0.601 | 0.13 (-0.66, 0.91) | 0.753 | -0.05 (-1.17, 1.07) | 0.931 |
| Hippocampal volume | 0.97 (-0.06, 2.00) | 0.064 | -0.04 (-0.99, 0.91) | 0.936 | -0.22 (-1.05, 0.62) | 0.604 |
| WMH volume | 0.25 (-0.65, 1.15) | 0.578 | -0.57 (-1.55, 0.40) | 0.245 | -1.13 (-2.51, 0.25) | 0.109 |

CI, confidence interval; SD: standard deviation; SPPB, Short Physical Performance Battery; WMH, white matter hyperintensities.

^1^ Cutoff of nutritional deficits: 25-hydroxyvitamin D <20 ng/ml, homocysteine >14 μmol/L, omega-3 index ≤ lower quartile (≤4.87%).

^2^ Adjustments for age, sex, Multidomain Alzheimer Preventive Trial (MAPT) groups, education, body mass index, physical activity status, total intracranial volume, brain volume (indicated in each row) and interactions between brain volume, nutritional deficits and time.

^3^ P-value indicates the interaction between nutritional deficits, brain volume and time.
